# The German System

**Published:** 1911-06-24

**Authors:** 


### THE GERMAN SYSTEM.

### I.—HOW STATE INSURANCE HAS HELPED THE GERMAN HOSPITALS.

By Our Special Commissioner.

The immense value of the legislative enactments with regard to invalidity, sickness, and accident insurance to the hospitals in Germany has been conclusively shown by Grotjahn in his interesting book,[1] and has been dealt with in a series of special articles in various magazines and journals.[2] There can be no reasonable doubt that the extensive development in hospital construction, management, and improvement generally which has been proceeding in Germany during the last two decades is directly due to the influence of these enactments upon the community at large. The member of the German Krankenkassen has learned to appreciate the value of hospital treatment; the fact that he has to pay for such treatment, and that he can, by gaining admission to a reputable hospital, secure the best that modern medicine and surgery can offer him, has largely conduced to rob the institutions of the stigma of pauperism that clung to them through the centuries, and that proved a perpetual stumbling block to the self-dependent worker who had his inevitable pride of class. It is now generally recognised that hospital treatment is imperatively necessary in the case of a member of a Krankenkas who cannot afford to pay for prolonged and costly home treatment, or who is, through private circumstances, unable to lie up in his own lodging, or who has need of better nursing and more skilled attendance than he can secure at home. At the same time it is increasingly felt that these desiderata are capable of considerable extension, and what Grotjahn calls the "Progressive Tendency of Hospitalisation in Germany" is really the result of organised effort to secure such amplification. By prevention more than by treatment of existing diseased conditions will the community be bettered in the long run; by isolating infectious cases and contacts, by treating disease in its earliest stage, and by enabling the worker to benefit to the full by modern improvements in prophylaxis and preventive medicine, the social and economic value of the citizen, and more especially the productive citizen, may be safeguarded. This is almost an admitted axiom, and it says much for the progressiveness of the German societies that they are fully alive to the importance of this aspect of the matter, and are doing their utmost, by the establishment of workers' sanatoriums, rest homes, and convalescent institutions, to make provision for the work that is to be done in the near future.

#### The Provisions of the Act.

The importance of hospital treatment in certain cases—for example, cases that need operation, not for the sake of convenience, but for the sake of urgency, such as acute abdominal conditions, cancers, trauma, etc.—is becoming increasingly appreciated by the community. It is a well-known fact that a patient is much better off in an up-to-date hospital, fully equipped with the best means for diagnosis and treatment, than at home or in a private nursing home where similar means, however excellent, are necessarily less ample and less easily available than in a public institution. While this is the case with the middle-class citizen, it is much more the case with the poor man. Thomalla[3] has tabulated the conditions under which it is imperative that the labourer should seek hospital aid; the novello to the German Sickness Insurance Act of 1903 gives the conditions under which a member of the Krankenkas is entitled to free treatment and nursing in a hospital as follows:—

"Whoever is married, or who has his own household, or is a member of a household, may, with his consent, or without his consent when the disease from which he suffers is an infectious disease, or when the nature of the disease is such that it does not admit of proper treatment or nursing being guaranteed by his family, or when his condition necessitates constant observation, or when he has persistently acted in contravention of the regulations already prescribed, be admitted into a hospital for such treatment.

"Other members, not falling under this section, may be taken to a hospital unconditionally.

"In all such cases the family of the patient is entitled to the half of the sick pay that may be granted to him so long as he is an inmate of such institution."

Under this section, as Grotjahn shows, it is obviously the society and not the doctor who judges whether a patient should go into a hospital. As a matter of fact, the decision has hitherto always rested with the physician in charge of the case. The theory that this right of the societies to cause a patient to be transferred to a hospital is a purely disciplinary measure, operating through the fear of the patient to be sent to a hospital, is no longer tenable when the hospital has gained so much in popularity that hospital abuse is no longer a fiction, but an admitted fact.

### The Fear of Hospital Treatment.

The sentence "when the patient has persistently acted in contravention of prescribed regulations," however, seems to show that the legislature had, when the Act was framed, some such disciplinary method in mind, but if that is the case it has long ago lost its force, and should be deleted. Under the German Act the insured person has no prescriptive right to hospital treatment—an omission due probably to the fact that the number of available beds at the time when the Act was passed was far less than the demands of the hospital population. There are one or two clauses in the Act which slightly compensate for this omission. For instance, section 21 says :—

"The period of sick pay may be extended for longer than twenty-six weeks to one year.

"Together with free treatment and nursing in a hospital, the insured may, in case he has relatives for whose support he has hitherto been responsible, be paid a sum not exceeding the half of his average daily rate of pay, such payment to be made to his family.

"Together with free treatment and nursing in a hospital, a patient who is an insured person under the Act, provided he has relatives for whose support he has not hitherto been responsible out of his wages, may be granted sick pay to the extent not exceeding a fourth of his average daily wage.

"For the period of one year from the date of the expiration of a patient's sick pay, provision may be made for convalescents by causing them to be admitted into a convalescent institution."

These provisions have materially affected the hospitals, since it has directly tended to increase the number of in-patients. A working man, under the Act, finds provision made for his family so long as he is an inmate of a hospital, while in addition he has the satisfaction of knowing that in case of prolonged convalescence his insuring society is to some extent responsible for him. The Act has provided for the building and equipment of proper convalescent institutions by the societies themselves, and many Krankenkassen have already availed themselves of these facilities, and by combining and co-operating have erected their own hospitals and homes—a tendency which is responsible for a great deal of discussion among German hospital authorities at the present moment.

### Some Interesting Statistics.

The following figures clearly show the amounts paid by the various societies during a number of years for the treatment of their members in hospitals. These figures, steadily increasing year by year, justify the conclusion that the development of hospitals in Germany during the last quarter of the nineteenth century has not only been of the greatest benefit to the community, but that on the other hand the increase in the number of persons who have availed themselves of the benefits under the Insurance Acts has been of material help towards the progressive development of the German hospital system.

Table Showing Amounts Paid by Insuring Societies Directly to Hospitals.

| Date | Total Amount paid in 1,000 marks | Amount paid per member (for hospital treatment only) | Amount paid per member (for medical treatment generally) |
|---|---|---|---|
| 1892 ... ... ... | 10125·0 | 1.50 | 13.55 |
| 1893 ... ... ... | 11569·0 | 1.63 | 14.35 |
| 1894 ... ... ... | 11868.8 | 1.63 | 13.67 |
| 1895 ... ... ... | 12604.1 | 1.67 | 13.93 |
| 1896 ... ... ... | 13575.0 | 1.71 | 13.81 |
| 1897 ... ... ... | 14804.8 | 1.78 | 14.45 |
| 1898 ... ... ... | 15852·3 | 1.81 | 14.60 |
| 1899 ... ... ... | 17883·8 | 1.95 | 15.87 |
| 1900 ... ... ... | 19607.8 | 2.06 | 16.58 |
| 1901 ... ... ... | 20641.2 | 2.14 | 16.95 |
| 1902 ... ... ... | 21343.0 | 2.16 | 17.02 |
| 1903 ... ... ... | 23658.8 | 2.31 | 17.69 |
| 1904 ... ... ... | 27694.3 | 2.59 | 19.97 |

Even more interesting than these totals is a comparison of the average expenditure of the Krankenkasse per member in case of sickness, and a glance at the tables published in the Statistik des Deutschen Reiches, Neue Folge, Bd. 170.[4] These tables were printed in full in Dr. H. P. Bosscha's article "Hospitals and State Insurance Systems," which appeared in The Hospital of October 1, 1910.

Every 100 marks expended by the sick funds was apportioned as follows :—

| Year | Doctor | Chemist | Sick Money | Convalescent Fund | Midwifery | Death | Hospital Treatment |
|---|---|---|---|---|---|---|---|
| 1888 | 20.34 | 16.16 | 47.11 | — | 1 32 | 4.27 | 10.80 |
| 1889 | 20.59 | 16.59 | 46.30 | — | 1.29 | 4.07 | 11.16 |
| 1890 | 19.97 | 16.88 | 47.46 | — | 1.21 | 3.90 | 10.58 |
| 1891 | 20.03 | 16.70 | 46.94 | — | 1.32 | 3.71 | 11.30 |
| 1892 | 20.23 | 17.02 | 46.63 | — | 1.29 | 3.77 | 11.06 |
| 1893 | 20.01 | 17.35 | 44.89 | 0.04 | 1.61 | 3.75 | 11.35 |
| 1894 | 22.30 | 17.50 | 42 78 | 0.07 | 1.78 | 3.65 | 11.92 |
| 1895 | 22.08 | 17.30 | 43.27 | 0.05 | 1.74 | 3.54 | 12.02 |
| 1896 | 22.61 | 17.23 | 42.35 | 0.07 | 1.84 | 3.53 | 12.37 |
| 1897 | 22.34 | 17.18 | 42.93 | 0.06 | 1.80 | 3.40 | 12.29 |
| 1898 | 22.73 | 17.19 | 42.47 | 0.07 | 1.83 | 3.33 | 12.38 |
| 1899 | 21.96 | 16.90 | 43.74 | 0.07 | 1.68 | 3.34 | 12.31 |
| 1900 | 21.75 | 16.47 | 44.31 | 0.07 | 1.62 | 3.36 | 12.42 |
| 1901 | 21.82 | 16.03 | 44.68 | 0 08 | 1.60 | 3.15 | 12.64 |
| 1902 | 22.35 | 15.84 | 44.33 | 0.08 | 1.62 | 3.05 | 12.72 |
| 1903 | 22.54 | 15.98 | 43 75 | 0 00 | 1 58 | 2.98 | 13.08 |
| 1904 | 22.40 | 15.02 | 44.77 | 0.07 | 2.00 | 2.79 | 12.95 |

From this and other tables it can be seen that the payments made by the sick funds to hospitals have been steadily increasing, while sick money, doctor and chemist bills, and especially death grants, have either declined or remained stationary.

In the year 1893 a sum of 11,568,966 marks was spent on cost of treatment in hospitals by the Krankenkassen; in 1904 it had risen to 27,694,385 marks, and the latest returns available show that it is steadily increasing at the same rate.

### The General Influence.

The importance of these figures is immense, and Grotjahn lays stress on the bearing which they have upon the hospital system generally.

"The hospitals," he remarks, "have lost their character as pauper asylums since the majority of their patients have been insured persons paying for their treatment. Since more and more cases are cured at hospitals, these institutions have gradually tended to become popular, and have lost their reputation as mere homes for the dying (Sterbehauser). The fear of hospitals, which still exists among a certain class of the community, is gradually being dissipated, and every year patients are becoming voluntary inmates. So popular indeed is hospital treatment becoming that the societies have seriously considered the advisability of building their own hospitals. That they have not done so yet on an extended scale, and have contented themselves with the establishment of homes for convalescents and infirm persons merely, is due to the decentralisation and want of co-operation that exist among them. It is necessary to guard against this tendency to provide independent individual institutions which may lead to an entirely unnecessary multiplication of hospitals, and consequently to a splitting of forces, by making the existing institutions perfect in every degree and by augmenting their resources, especially by adding to their special departments, so that they are able to deal with every case that comes to them."

By the Accident Insurance Act of 1884, amplified by the novello of 1900, an insured person, in case of accident, may, under certain conditions, elect to be treated in hospital. According to Thiem,[5] the choice of hospital or private treatment rests with the insuring society. If the patient refuses to enter a hospital when ordered to do so, or leaves the institution before he is allowed to do so (except when good reason can be shown for such action, as, for instance, it has been held that a patient is entitled to go home in case his presence is absolutely required through his wife's confinement), the accident pay may be withdrawn or decreased. The figures quoted in Grotjahn show how generously the societies have contributed towards the funds of hospitals for the treatment of their members who have been injured or incapacitated through accident, but are too long to be quoted here.

### Invalidity Insurance.

A still wider influence has been exerted on hospitals in Germany by the Invalidity Insurance Act. This has been responsible primarily for the popular interest in sanatorium treatment. Under this Act the societies are empowered to provide for the treatment of incapacitated persons insured by them in proper institutions, and to pay invalidity pay to their families under certain conditions. In this connection again the figures quoted are interesting. We can only give here the totals; for the full list, extending over several pages, we must refer readers to Dr. Grotjahn's book. In the year 1905 out of 11,813,259 insured persons, 26,621 persons were treated at various institutions for commencing tuberculosis (phthisis only). Out of this grand total no less than 21,788 were cured in the sense that they were no longer "incapacitated" under the Act, while 4,833 were so incapacitated. The cost to the societies was 9,679,535 marks, or a sum of 394 marks for every cured and 225 marks for every incapacitated person. In the same year 22,322 insured persons were treated for other forms of tuberculosis, at a cost of 4,483,192 marks; of these 18,265 were cured and 8,057 returned as "Erwerbsunfähig." As a result of the Act the number of sanatoria, both private and public, has increased enormously during the last ten years; individual societies have built their own institutions (as in this country at Benenden), and there is now a tendency, which is to be encouraged, to combine and co-operate with the object of establishing communal sanatoriums or large district institutions where more patients can be treated under better conditions than at the smaller sanatoriums.

Grotjahn's final conclusions are worth giving in full, and we make no apology for inserting them here, as they have a practical interest at the present juncture :—

1. The social insurance legislation has very greatly influenced the development of the hospital system, and has made hospital treatment popular in Germany.
2. The Sickness Insurance Act has specially contributed to the increase and improvement of general hospitals, and have deprived these of their character as pauper asylums.
3. The Accident Insurance Act has stimulated the increase and improvement of the surgical departments of general hospitals, and has forced these to pay attention not only to the surgical but to the functional cure of injured patients.
4. The Invalidity Insurance Act has called into being an entirely new kind of hospital treatment, the preventive treatment of disease.

---

[1] Dr. Alfred Grotjahn, "Krankenhauswesen und Heilstättenbewegung." Leipsig : F. Vogel. 1908.

[2] It is impossible for us to give full references to the German literature on the subject. A condensed bibliography is to be found in the *Jahresbericht über Soziale Hygiene* (which itself contains many instructive papers), while excellent informative articles have appeared in the *Zeitschrift für Soziale Medizin.* The standard bibliography has been compiled by Roth, for the Liebe-Jacobsohn-Meyer "Textbook of Hospital Management." A large number of special articles have been consulted in drawing up these summaries, and in particular we wish to express our indebtedness to the statistical and other reports emanating from the Reichsversicherungsamt at Berlin. In the circumstances it has seemed to us to be unnecessary to devote a special article to the bibliography and references.

[3] Ueber die Behandlung erkrankter Kassenmitglieder, 1894.

[4] "Statistik der Krankenversicherung im Jahre 1904." Berlin : Puttkammer u. Mühlbrecht. 1907.

[5] C. Thiem, "Spezielle Krankenversorgung für Arbeiter." 1899.

